# The Role of Toll-like Receptor 2 (TLR2) in the Development and Progression of Hashimoto’s Disease (HD): A Case Study on Female Patients in Poland

**DOI:** 10.3390/ijms24065344

**Published:** 2023-03-10

**Authors:** Maria Klatka, Agnieszka Polak, Paulina Mertowska, Sebastian Mertowski, Jakub Łyczba, Anna Hymos, Izabela Korona-Głowniak, Ewelina Grywalska

**Affiliations:** 1 Department of Pediatric Endocrinology and Diabetology, Medical University of Lublin, 20-093 Lublin, Poland; 2 Department of Endocrinology, Medical University of Lublin, 20-093 Lublin, Poland; 3 Department of Experimental Immunology, Medical University of Lublin, 20-093 Lublin, Poland; 4 Department of Pharmaceutical Microbiology, Medical University of Lublin, 20-093 Lublin, Poland

**Keywords:** Hashimoto’s disease, dendritic cells, monocytes, Toll-like receptors, TLR2

## Abstract

Chronic lymphocytic thyroiditis, commonly known as HD, is one of the most common thyroid disorders. Due to the diverse factors affecting the etiopathogenesis of this disease (hormonal disorders and genetic and environmental factors), as well as the direct involvement of the immune system, scientists are increasingly willing to undertake research aimed at explaining the impact of the loss of immune tolerance and reactivity of autoantigens on the development of the disease. One of the directions of research in recent years is the role of the innate immune response, particularly Toll-like receptors (TLRs), in the pathogenesis of HD. The purpose of this study was to determine the importance of Toll-like receptor 2 (TLR2) expression on selected populations of immune cells, namely, monocytes (MONs) and dendritic cells (DCs), in the course of HD. Particular attention was paid to the analysis of TLR2’s correlation with clinical parameters and the possibility its use as a potential biomarker molecule in the diagnostic process. Based on the obtained results, we found a statistically significant increase in the percentage of all analyzed populations of immune cells, i.e., mDC BDCA-1+CD19−, pDC BDCA-1+CD123, classical MONs CD14+CD16−, and non-classical MONs CD14+CD16+ showing on their surface TLR2 expression in patients diagnosed with HD compared to the healthy volunteers. Moreover, in the study group, we noted a more than 6-fold increase in the concentration of the soluble form of TLR2 in plasma compared to healthy patients. In addition, the correlation analysis showed significant positive correlations between the level of TLR2 expression on selected subpopulations of immune cells and biochemical indicators of thyroid function. Based on the obtained results, we can assume that TLR2 may be involved in the immunopathogenesis of HD.

## 1. Introduction

According to literature data and research reports, chronic lymphocytic thyroiditis, or HD, is considered one of the most common causes of hypothyroidism in modern society [1,2]. Epidemiologically, this disease affects women more often than men, and, according to the latest studies, its incidence varies not only in terms of gender, but also in terms of the geographical location, region, and socio-economic status of its inhabitants. According to literature reports, HD can affect up to 4.8–25.8% of women and 0.9–7.9% of men [3,4,5]. Studies show that the frequency of developing this disease increases with the age of patients (especially after 60 years of age); however, more and more often, this entity is also diagnosed in young people and even children [6,7,8,9]. The causes of hypothyroidism and thus the development of HD are many, ranging from genetic factors and hormonal changes to environmental factors, as well as the presence of other comorbidities, especially those of autoimmune origin [10,11,12,13]. HD itself is classified as a subunit of autoimmune diseases, which means that its development is closely related to the deregulation of the immune system, and, more specifically, to the development of inflammation caused by the host’s immune cells (in particular T lymphocyte-induced apoptosis of thyroid follicular cells) that attack the thyroid [14,15,16]. The consequence of this is the gradual destruction of this gland, leading to disorders in the production of triiodothyronine (T3) and thyroxine or tetraiodothyronine (T4) hormones, as well as the thyroid-stimulating hormone (TSH) that controls them [17,18,19,20]. Thyroid hormones are responsible for the induction of many reactions important for the immune system, such as the proliferation and migration of leukocytes, as well as the release of cytokines or the production of antibodies necessary for a proper immune response [21,22,23,24]. Despite research on the relationship between thyroid hormones and the immune system, the reasons for the development and progression of HD remain ambiguous. An increasing number of studies have focused on identifying abnormalities related to the immune system, particularly in the mechanisms of the innate immune response, which, researchers believe, can have a significant impact on the development of autoimmune thyroid diseases (AITD) [25,26,27,28,29]. One of the subjects of in-depth research is the participation of TLRs, responsible for the recognition of damage-associated molecular patterns (DAMPs) and pathogen-associated molecular patterns (PAMPs) [30,31,32,33]. These receptors can stimulate many of the cells of the immune system, including the production of monocytes (MONs) and antigen-presenting cells, by activating signaling pathways leading to the release of inflammatory mediators [25,33,34,35]. One of the subjects of our research is TLR2, which is a surface protein expressed on numerous cells of the immune system, such as MONs, macrophages, dendritic cells (DCs), and T and B lymphocytes [36,37]. This receptor recognizes many substances of bacterial, fungal, and viral origin, as well as some endogenous substances [37,38,39]. The purpose of this research was to determine the importance of TLR2 expression on selected populations of immune cells, specifically MONs and DCs, during HD. Particular attention was paid to the analysis of the TLR2 correlation with clinical parameters and the possibility of using it as a potential biomarker molecule in the diagnostic process.

## 2. Results

### 2.1. Analysis of Selected Parameters of Peripheral Blood Morphology and Thyroid Function in Patients with HD and Healthy Volunteers

The study included 35 women with newly diagnosed HD and 20 healthy women. The analysis of the basic parameters of peripheral blood (PB) showed a significant reduction in the level of lymphocytes by 30.58% in Hashimoto’s patients compared to the control group (Table 1). Other parameters of blood counts did not differ significantly between the tested groups, and their values ranged within laboratory standards. Biochemical analyses of the peripheral blood were carried out for the selected indicators of thyroid function, which showed a drastic decrease in TSH levels (267-fold) and an increase in the free fraction of triiodothyronine (fT3) (4.40-fold) and free fraction of thyroxine or tetraiodothyronine (fT4) (2.08-fold) in patients from the study group in relation to control group (Table 1). 

### 2.2. Characteristics of the Percentage of Selected Populations of Immune Cells Expressing TLR2 and Soluble Toll-like Receptor 2 (sTLR2) Concentration in the Plasma of Patients with HD in Relation to the Control Group

In the next stage of the study, we performed an immunophenotypic analysis of the percentage of the occurrence of two subpopulations of DCs, i.e., myeloid dendritic cells (mDC) and plasmacytoid dendritic cells (pDCs), as well as classical and non-classical MONs. The first DCs’ subpopulation to be studied was mDC, which is classified as antigen presenting cells (APCs), which participate in the process of capturing, processing, and presenting antigens on their surface to T lymphocytes. In the available scientific literature, mDC cells are considered as a kind of link between the innate and acquired immune response [40,41]. The second DC subpopulation analyzed was pDCs, which are a unique subset of cells specialized in secreting high levels of type I interferons and are involved in the initiation and development of many autoimmune and inflammatory diseases [42,43]. In the case of MONs, which are an important part of the innate immune system, in cytometric studies, we can distinguish at least three subclasses found in human blood based on their phenotypic receptors [44]. In our analysis, we conducted studies on classical MONs CD14+CD16− and non-classical MONsCD14+CD16+.

The analyses show that patients with HD have a higher percentage of mDC BDCA-1CD19− (by 88.88%) and pDC BDCA-2+CD123+ (by 113.04%), as well as a decrease in the percentage of classical MONs CD14+CD16− (by 7.84%) in PB compared to patients in the control group (Table 2).

The next step of our research was to assess the percentage of the occurrence of the individual subpopulations of the tested immune system cells expressing TLR2 on their surface. In all studied cases, the percentage of the individual cells of the immune system expressing TLR2 was statistically and significantly higher in patients with HD compared to the control group: for mDC BDCA-1+CD19−TLR2+ (3.99-fold average); for pDC BDCA-2+CD123+TLR2+ (average 3.14-fold); for classical MONs CD14+CD16−TLR2+ (mean 3.67-fold); for non-classical MONs CD14+CD16+TLR2+ (mean 3.45-fold) (Table 2).

The final stage of the laboratory tests included the assessment of the concentration of the sTLR2 in the plasma of both groups of patients classified for the study. The conducted experiment showed that patients with HD have a 6.45-times higher concentration of sTLR2 in plasma compared to patients from the control group (Table 2).

### 2.3. Correlation Analysis of the Percentage of Tested Immune System Cells Expressing TLR2 and sTLR2 Concentration in Relation to Selected Biochemical Indices Determining the Functioning of the Thyroid Gland

The statistical analysis aimed to determine whether there are any specific correlations between the level of TLR2 expression on the selected cells of the immune system and biochemical parameters indicating abnormal thyroid function in patients with HD. For this purpose, we analyzed in detail all the relationships between the percentage of the occurrence of the individual subpopulations of DCs and MONs expressing TLR2 among themselves and in relation to the level of TSH and the concentration of fT3 and fT4 and the concentration of sTLR2 in the plasma of patients.

**Figure 1:**
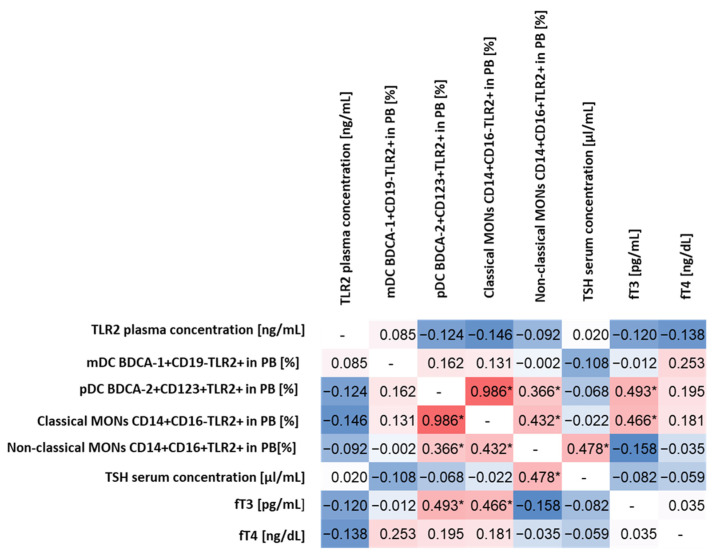
Spearman’s rank correlation analysis of the percentage of TLR2-expressing DCs and MONs and the concentration of sTLR2 in relation to selected biochemical indices determining the functioning of the thyroid gland. The diagram shows the correlations in red (the intensity of the color indicates the strength of the correlation), while the negative correlations are marked in blue (the intensity of the color indicates the strength of the correlation). Identical pairs of parameters that were not correlated during the analysis were marked in white with the “-” sign. * Statistically significant results.

The conducted analysis allowed to observe statistically significant correlations between pDC BDCA-2+CD123+TLR2+ and fT3 concentrations (moderate positive correlation) and classical MONs CD14+CD16−TLR2+ (high positive correlation) and non-classical MONs CD14+CD16+TLR2+ (low positive correlation) concentrations (Figure 1). For classical MONs CD14+CD16−TLR2+, moderately positive correlations were observed for non-classical MONs CD14+CD16+TLR2+ and fT3 concentration, while, for non-classical MONs CD14+CD16+TLR2+ for the TSH level (Figure 1), we did not observe statistically significant correlations between the expression of TLR2 on the tested subpopulations of immune system cells and the concentration of sTLR2 in plasma, as well as between its concentration and biochemical parameters of thyroid function.

### 2.4. Analysis of the Potential of TLR2 in the Context of Diagnostic Accuracy in Differentiating Patients with HD

**Figure 2:**
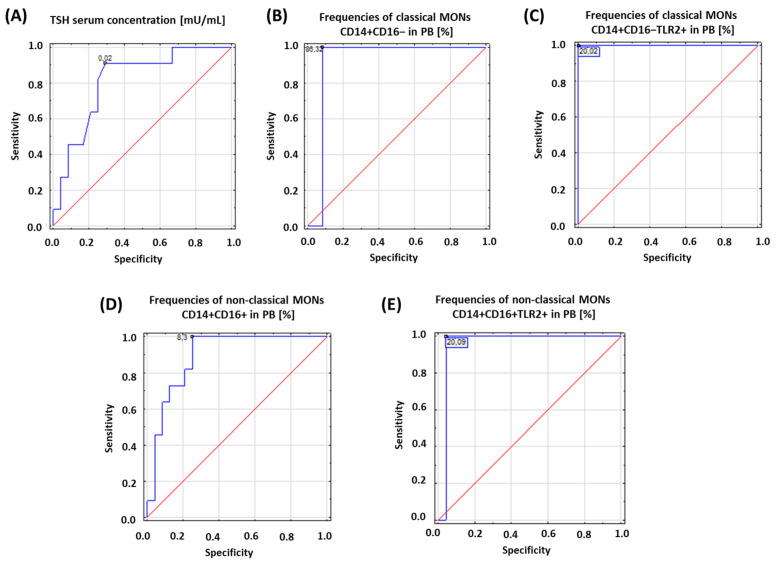
Schematic representation of the results obtained from ROC analysis to determine diagnostic accuracy of patients with HD: (**A**) ROC analysis for TSH serum concentration; (**B**) ROC analysis for frequencies of classical MONs CD14+CD16− in PB [%]; (**C**) ROC analysis for frequencies of classical MONs CD14+CD16−TLR2+ in PB [%]; (**D**) ROC analysis for frequencies of non-classical MONs CD14+CD16+ in PB [%]; (**E**) ROC analysis for frequencies of non-classical MONs CD14+CD16+TLR2+ in PB [%].

Based on the obtained results, we assessed the diagnostic accuracy of selected analyzed parameters based on receiver operating characteristic (ROC) curves. The concentration of TSH and the percentage of the occurrence of both subpopulations of MONs expressing TLR2 on their surface were analyzed. The obtained results are presented in Table 3 and Figure 2.

## 3. Discussion

The increase in the number of autoimmune diseases observed in recent years has caused many scientists to attempt to explain the role of the immune system, and in particular the deregulation of its functioning, in the development and progression of this unusual group of diseases. Disorders in the endocrine system are more and more common in modern society, preventing the proper functioning of the body and, in some cases, posing a direct threat to life [45,46,47]. Hypothyroidism is the most common thyroid disorder affecting both men and women worldwide, and it also affects the proper functioning of the immune system [48,49,50,51]. According to the literature, the number of cases of hypothyroidism caused by HD has been steadily increasing in recent years. During HD, the immune system produces antibodies against thyroid peroxidase (aTPO), an enzyme involved in the proper synthesis of thyroxine—one of the thyroid hormones. These antibodies attack the thyroid gland and destroy it, leading to hypothyroidism [52,53,54]. In addition, according to scientists, the development of AITDs, which undoubtedly include HD, occurs as a result of the loss of immune tolerance, accompanied by increased reactivity to thyroid autoantigens [55,56,57,58,59,60]. In addition, studies indicate that, in the phase of induction and effector immune and inflammatory response, and playing a significant role in the immunopathogenesis of AITDs, cytokines secreted by cells of the immune system are also involved, such as Interleukin-1 alpha (IL-1α), Interleukin-1 beta (IL-1β), IL-2, IL-4, IL-6, IL-8, IL-10, IL-12, IL-13, IL-14, or tumor necrosis factor alpha (TNF-α) [61,62,63]. According to researchers, the mechanisms of the disorders of the immune system are not only involved in the pathogenesis of AITD, but are also strongly interrelated; however, the differences in the phenotypes of the individual disease subunits require intensive research. Thanks to the development of molecular methods, changes that occur during the development and progression of HD in the context of deregulation of the immune system have begun to be looked at a little closer [1,64,65]. Most literature reports have focused on and considered the abnormalities occurring during the activation of the innate immune response as one of the mechanisms involved in the pathogenesis of all AITDs. Toll-like receptors, as one of the most conservative molecules that bridge the innate and adaptive immune responses, have become the main focus of research [26,28]. Due to their ubiquitous presence in the cells of the immune system and involvement in cell signaling pathways, they have become an element which combines our existing knowledge on the potential causes of the development of autoimmune diseases. Of the more than 1600 articles published over the past 23 years regarding the role of TLRs in the development of autoimmune diseases, only 53 of them concern the involvement of these unusual receptors in the pathogenesis of HD. The available research data emphasize that TLRs can have a significant impact on the deregulation of the immune system and thus contribute to the development of HD.

Haria’s team in 2005 showed that TLR3 is highly expressed in the thyrocytes of patients with Hashimoto’s disease, which, according to the researchers, may affect the mechanisms of the innate immune response in these cells. This is important not only in the pathogenesis of HD, but also in the process of the infiltration of immune cells [66].

The research conducted by the Peng team in 2016 showed that the messenger RNA (mRNA) expression level of the tested TLR2, TLR3, TLR9, and TLR10, in the peripheral blood mononuclear cell (PBMC) of patients with AITD, was significantly increased compared to a control group [67]. Moreover, the surface expression of TLR2, TLR3, and TLR9 was increased in monocytes from AITD patients compared to controls [67]. According to scientists, this confirms that TLRs may be involved in the pathogenesis of AITD; however, their role remains unclear. These results confirm our observations, which show that the percentage of both monocyte subpopulations expressing TLR2 was higher in patients with HD compared to the healthy volunteers, by 3.67 times and 3.45 times, respectively. Moreover, these changes also concerned the analyzed subpopulations of DCs, for which mDC BDCA-1+CD19−TLR2+ and pDC BDCA-2+CD123+TLR2+ were higher on average by 3.99 times and 3.14 times compared to healthy patients.

The Aktaş team, in 2020, showed that the serum levels of TLR2 and TLR4 in healthy volunteers were higher than in patients with HD [68]. This is identical to the results obtained by our team, where the sTLR2 concentration was more than six-times higher in patients from the study group compared to the control group. Consistent with the presented study results, the authors concluded that TLR2 and TLR4 may be involved in the immunopathological mechanisms of HD by potentiating the induction of proinflammatory responses in these patients.

Further studies available in the literature focus on the role of the link between single nucleotide polymorphisms (SNPs) and the development of HD, which scientists believe may affect the expression of proinflammatory cytokines and chemokines, as well as the production of transcription factors, either directly or indirectly, though through signaling pathways involving TLRs. However, there are very few such studies, and the presented data are often insufficient to draw clear conclusions due to too small a sample [69].

In 2017, Cho’s team conducted polymorphism studies for TLR1–TLR6 and TLR9 genes to identify possible associations between the development of autoimmune thyroid disease (Hashimoto’s and Graves’ disease) and Korean children [70]. Based on their analyses, they found 15 SNPs: one SNP found in TLR1 and TLR5; two SNPs for TLR3 and TLR6; and three SNPs for TLR2, TLR4, and TLR9. However, the frequency of the identified genotypes except for TLR4 and TLR9 was not statistically significant [70].

In 2020, the Aktaş team analyzed the genotype frequency and allele frequency distribution of TLR2 and TLR4 gene polymorphisms; however, the changes observed between the study group and the control group were not statistically significant [69].

The available literature data on the involvement of TLRs in the pathogenesis of HD are scarce and still require many intensive studies to clarify their role in detail.

## 4. Materials and Methods

### 4.1. Characteristics of Patients and Control Groups

The studied group included 35 women with newly diagnosed HD, who were treated in the Clinic of Endocrinology of the Medical University and Clinics of Endocrinology Independent Public Clinical Hospital No. 4 (SPSK-4) in Lublin. The control group consisted of 20 healthy women hospitalized at the Department of Otolaryngology of the Medical University of Lublin due to nasal septum deviation and external deformities of the nose, with normal thyroid function, which was confirmed using hormonal tests (Table 4). The criteria for the exclusion of patients from the study included the use of drugs that affect the functioning of the immune system; reported symptoms of infection in the last 4 weeks before the study; patients with diagnosed allergies as well as concomitant autoimmune diseases; blood transfusion procedure. The diagnosis of hyperthyroidism was based on clinical features and laboratory markers of hyperthyroidism (Table 4).

The studied material was peripheral blood from patients in the amount of 15 mL collected to the tube with anti-coagulant ethylenediaminetetraacetic acid (EDTA) (to obtain plasma and cells from peripheral blood) during routine laboratory examinations. Every patient expressed a conscious written consent for participating in this study. The Bioethical Commission at the Medical University in Lublin (EC Resolution No. KE-0254/191/2018) gave appropriate permission to conduct this research.

### 4.2. The Assessment of the Population of DC and Monocytes in Peripheral Blood

**Figure 3:**
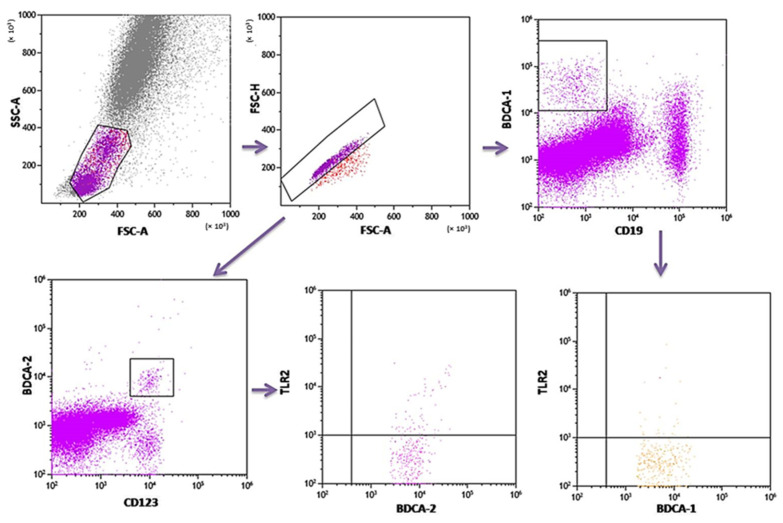
Sample analysis of the percentage of PB DCs expressing TLR2.

**Figure 4:**
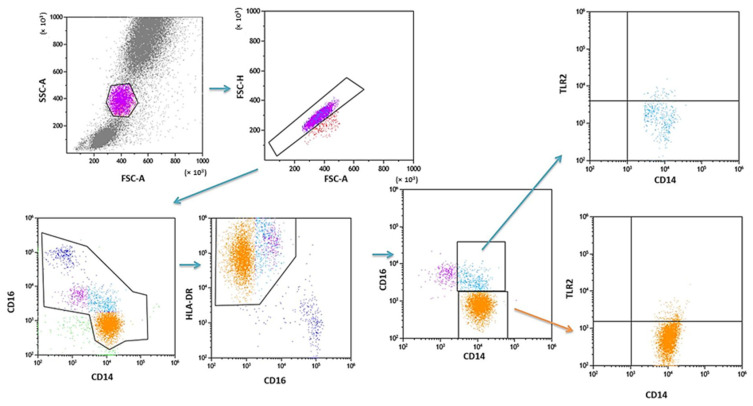
Sample analysis of the percentage of PB MONs expressing TLR2.

PB was diluted with 0.9% phosphate-buffered saline (PBS) without Ca^2+^ Mg^2+^ ions (Biochrome AG, Berlin, Germany) in a 1:1 and 1:2 ratio. The diluted material was layered on Lymphoprep (Axis-Shield PoC AS, Göteborg, Sweden), with a specific weight of 1.077 g/mL. Then, samples were centrifuged in a density gradient at 700× *g* for 20 min at 21 °C. Isolated cells were collected with Pasteur pipettes and washed twice in PBS (centrifugation 300× *g* for 5 min in 21 °C). To exclude the unspecific binding of antibodies, the Fc receptor was inactivated by incubation of cells with 20 μL of blocking buffer (FcR—Blocking Reagent, Miltenyi Biotec, Bergisch Gladbach, Germany). Then, the cell suspension was split into tubes into the amount of 1 × 10^7^ cells, with 100 μL of PBS in each tube. To assess the percentage of DC, the cells were subjected to a staining procedure with monoclonal antibodies conjugated with fluorochromes such as fluorescein isothiocyanate (FITC), CyChrome, and phycoerythrin (PE). The following antibodies were used: IgG1 FITC/IgG2a R-PE/IgG2a PE-Cy5 (Caltag Medsystems Ltd, Buckingham, UK) (negative control for green, orange and red fluorescence); anti-CD45 FITC/anty-CD14 R-PE (isotype: IgG1/IgG2a) (Caltag Medsystems Ltd, Buckingham, UK) (to precisely determine the gate for the population of mononuclear cells); anti-BDCA-1 FITC/anty-CD19 PE-Cy5 (isotype: IgG1/IgG1) (Miltenyi Biotec, Niemcy/BD Pharmingen, New Jersey, USA) (to determine the percentage of immature DCs from myeloid cell line); anti-BDCA-2 FITC/anty-CD123 PE (isotype: IgG1/IgG1) (Miltenyi Biotec, Niemcy/Becton Dickinson, San Diego, CA, USA) (to determine the percentage of immature DCs from lymphoid cell line). Next, 10 μL of antibodies was added to 100 μL of PB and incubated at 4 °C for 10 min in dark. Next, the cells were washed with PBS, containing 0.5% bovine serum albumin (BSA) and 0.2 mM EDTA, and centrifuged for 10 min at 300× *g* in 4 °C. After incubation, the cells were washed twice with PBS, centrifuged at 700× *g* for 5 min, and then immediately analyzed in a FACSCalibur flow cytometer (Becton Dickinson, San Diego, CA, USA). Data acquisition was performed using the Fluorescence activated cell sorting (FACS) Diva Software 6.1.3 software, collecting 30,000 cells for each assay, while analysis was performed using the CellQuest Pro program (Becton Dickinson, San Diego, CA, USA). An example of the cytometric analysis of assessing populations of BDCA-1+ and BDCA-2+ DC is shown in Figure 3. The percentage of classical MONs (CD14+CD16−) and non-classical MONs (CD14+CD16+) expressing TLR2 was measured using fluorochrome-conjugated mouse monoclonal antibodies: anti-CD14 FITC (BD Biosciences, San Jose, CA, USA), anti-CD16 V450 (BD Biosciences, San Jose, CA, USA), anti-HLA-DR Pe-Cy7 (BD Biosciences, San Jose, CA, USA), anti-TLR2 PE (Biolegend, San Diego, CA, USA), and 100 μL whole PB. An additional gating step with HLA-DR was used to improve monocyte purity. An example od the cytometric analysis is presented in Figure 4.

### 4.3. Quantification of the sTLR in the Plasma of Patients by Enzyme-Linked Immunosorbent Assay (ELISA)

The commercial ELISA kit Human Toll Like Receptor 2 (TLR2) (MyBioSource, San Diego, CA, USA), with a sensitivity less than 0.078 ng/mL and an assay range of 0.312–20 ng/mL, was used for the quantitative determination of human TLR2 in plasma samples. Plasma collected from EDTA samples was stored in liquid nitrogen until analysis. The analysis was performed in accordance with the manufacturer’s recommendations. The ELISA Reader VictorTM3 (PerkinElmer, Waltham, MA, USA) microplate reader was used for measurements. According to the manufacturer’s instructions, the determination was carried out at a wavelength of 450 nm.

### 4.4. Statistical Analysis

Obtained results were subjected to statistical analysis. The values of the analyzed parameters were characterized using arithmetic mean, standard deviation (SD), and median with variability range. The Mann–Whitney U test was used for intergroup comparisons. The power and direction of relationships between pairs of continuous variables were determined using Spearman’s coefficient of rank correlation. The differences were considered significant at *p* < 0.05. Statistical analyses were performed using Statistica v. 13.0 (StatSoft) software.

## 5. Conclusions

The results of our research presented in this publication suggest that TLR2 may significantly influence the dysregulation of the functioning of the immune system and thus the development and progression of Hashimoto’s disease. This is due to the increased percentages of both dendritic cells and monocytes expressing TLR2 on their surface in patients with Hashimoto’s disease in relation to the control group, as well as a more than six-fold increase in the concentration of sTLR2 in the plasma of the patients. The presented correlations with the selected biochemical indices of thyroid function inspire optimism for further in-depth research. Due to the limited number of patients enrolled in the study, it is important to conduct further interdisciplinary studies to clarify the exact role of TLR2 in the immunopathogenesis of Hashimoto’s disease. We hope that the presented data also encourage other scientists to develop this topic and make a joint effort to explain the role of TLRs in the pathogenesis of autoimmune thyroid diseases.

## Figures and Tables

**Table 1:** Comparison of selected PB count parameters and thyroid function indicators in patients diagnosed with HD in relation to patients in the control group.

Parametrs	Patients with HD (n = 35)		Healthy Volunteers (n = 20)		*p*-Value
	Mean ± SD	Median (Range)	Mean ± SD	Median (Range)	
WBC [10^3^/mm^3^] (Normal Range: 4.37–10.00)	6.15 ± 2.05	6.38 (2.53–10.62)	6.49 ± 2.01	6.11 (4.19–10.62)	0.815
LYM [10^3^/mm^3^] (Normal Range: 1.18–3.74)	1.68 ± 0.60	1.63 (0.76–3.27)	2.42 ± 0.48	2.29 (1.29–3.42)	0.000 *
MON [10^3^/mm^3^] (Normal Range: 0.24–0.63)	0.52 ± 0.19	0.49 (0.20–0.82)	0.50 ± 0.15	0.44 (0.25–0.82)	0.633
NEU [10^3^/mm^3^] (Normal Range: 1.56–6.13)	3.49 ± 1.38	3.59 (0.91–5.47)	3.45 ± 1.18	3.22 (1.59–5.47)	0.842
EOS [10^3^/mm^3^] (Normal Range: 0.04–0.36)	0.13 ± 0.07	0.12 (0.05–0.43)	0.12 ± 0.09	0.10 (0.04–0.43)	0.450
BAS [10^3^/mm^3^] (Normal Range: 0.01–0.08)	0.04 ± 0.02	0.03 (0.00–0.06)	0.04 ± 0.02	0.03 (0.01–0.06)	0.951
RBC [10^6^/mm^3^] (Normal Range: 3.93–5.22)	4.55 ± 0.31	4.58 (3.91–5.66)	4.45 ± 0.49	4.47 (3.81–5.66)	0.095
HGB [g/dL] (Normal Range: 11.20–15.70)	13.34 ± 0.91	13.30 (11.10–14.70)	13.08 ± 0.96	13.20 (11.60–14.70)	0.609
PLT [10^3^/mm^3^] (Normal Range: 150.00–400.00)	291.86 ± 73.27	299.00 (128.00–387.00)	261.55 ± 52.03	246.00 (182.00–387.00)	0.126
TSH [µL/mL] (Normal Range: 0.30–3.00)	0.01 ± 0.01	0.01 (0.00–0.09)	2.67 ± 0.80	2.71 (0.92–3.98)	0.000 *
fT3 [pg/mL] (Normal Range: 2.00–4.40)	11.75 ± 5.14	11.14 (6.90–31.79)	4.98 ± 0.87	4.97 (3.60–6.40)	0.000 *
fT4 [ng/dL] (Normal Range: 0.93–1.70)	35.49 ± 16.02	30.95 (19.05–82.22)	17.09 ± 2.39	17.04 (11.47–21.47)	0.000 *

Abbreviations: WBC—white blood cells; LYM—lymphocytes; MON—monocytes; NEU—neutrophils; BAS—basophils; EOS—eosinophils; RBC—red blood cell count; HGB—hemoglobin; PLT—platelets; TSH—thyrotropic hormone; fT3—free fraction of triiodothyronine; fT4—free fraction of thyroxine; * Statistically significant results.

**Table 2:** Comparison of the percentage of DCs and MONs expressing TLR2 and sTLR2 concentration in the plasma of patients with Hashimoto in relation to patients in the control group.

Parametrs	Patients with HD (n = 35)		Healthy Volunteers (n = 20)		*p*-Value
	Mean ± SD	Median (Range)	Mean ± SD	Median (Range)	
mDC BDCA-1+CD19− in PB [%]	1.02 ± 0.36	1.02 (0.44–1.82)	0.54 ± 0.29	0.53 (0.13–1.10)	0.0000 *
pDC BDCA-2+CD123+ in PB [%]	0.49 ± 0.36	0.40 (0.02–1.54)	0.23 ± 0.24	0.15 (0.01–0.97)	0.0000 *
mDC BDCA-1+CD19−TLR2+ in PB [%]	20.12 ± 7.60	20.20 (7.50–35.40)	5.04 ± 3.49	4.30 (1.30–13.70)	0.0000 *
pDC BDCA-2+CD123+TLR2+ in PB [%]	13.33 ± 6.12	12.60 (5.90–27.80)	4.24 ± 2.93	3.60 (1.40–11.50)	0.0004 *
Classical MONs CD14+CD16− in PB [%]	85.80 ± 4.70	87.10 (75.50–94.50)	93.10 ± 2.40	95.5 (88.30–95.40)	0.028 *
Non-classical MONs CD14+CD16+ in PB [%]	9.9 ± 4.9	8.5 (3.1–20.5)	4.4 ± 2.2	3.6 (2.2–10.3)	0.2200
Classical MONs CD14+CD16−TLR2+ in PB [%]	15.9 ± 12.9	11.9 (2.5–43.4)	4.33 ± 1.96	4.00 (2.00–9.10)	0.0003 *
Non-classical MONs CD14+CD16+TLR2+ in PB [%]	18.42 ± 14.85	15.80 (1.10–50.80)	5.34 ± 2.73	4.80 (1.80–9.90)	0.0007 *
TLR2 plasma concentration [ng/mL]	32.19 ± 13.34	30.4 (14.40–56.90)	4.98 ± 3.87	4.6 (0.7–13.9)	0.000 *

Abbreviations: TLR2—Toll-like receptor 2; * Statistically significant results.

**Table 3:** ROC analysis to determine diagnostic accuracy of patients with HD.

Parameter	Prognostic Value	Area under the ROC Curve (AUC)	95% Confidence Interval (95%CI)
TSH serum concentration [µL/mL]	0.02	0.81	0.66–0.96
Frequencies of classical MONs CD14+CD16− in the PB [%]	86.32	0.92	0.81–1.0
Frequencies of classical MONs CD14+CD16−TLR2+ in the PB [%]	20.02	1.0	1.0
Frequencies of non-classical MONs CD14+CD16+ in the PB [%]	8.3	0.89	0.79–0.998
Frequencies of non-classical MONs CD14+CD16+TLR2+ in the PB [%]	20.09	0.96	0.88–1.0

**Table 4:** Characteristics of the studied group (n = 35).

Parametrs	Patients with HD (n = 35)		Healthy Volunteers (n = 20)	
	Mean ± SD	Median (Range)	Mean ± SD	Median (Range)
Age (years)	41.00 ± 16.21	36.00 (22.00–95.00)	39.30 ± 11.30	36.00 (23.00–65.00)
TSH Receptor Antibodies (TRAb) [U/L]	12.17 ± 9.69	9.75 (1.70–39.40)	1.65 ± 0.69	1.45 (0.59–1.86)
anti-TPO antibodies [U/mL]	1272.36 ± 551.83	1322.70 (13.70–1873.70)	1.69 ± 0.31	1.54 (0.74–4.51)
anti-TG antibodies [U/mL]	255.81 ± 292.77	160.77 (10.00–1360.00)	1.31 ± 0.27	1.58 (0.89–3.11)
Thyroid gland volume [mL]	20.47 ± 8.32	20.35 (13.30–47.60)	not applicable	
fT3 [pg/mL]	11.75 ± 5.14	11.14 (6.90–31.79)	4.98 ± 0.87	4.97 (3.60–6.40)
fT4 [ng/dL]	35.49 ± 16.02	30.95 (19.05–82.22)	17.09 ± 2.39	17.04 (11.47–21.47)
TSH [µL/mL]	0.01 ± 0.01	0.01 (0.00–0.09)	2.67 ± 0.80	2.71 (0.92–3.98)

## Data Availability

Access to data at the request of the person concerned, by contacting the corresponding author.
